# Pre-Targeting and Direct Immunotargeting of Liposomal Drug Carriers to Ovarian Carcinoma

**DOI:** 10.1371/journal.pone.0041410

**Published:** 2012-07-26

**Authors:** Julia Lehtinen, Mari Raki, Kim A. Bergström, Päivi Uutela, Katariina Lehtinen, Annukka Hiltunen, Jere Pikkarainen, Huamin Liang, Sari Pitkänen, Ann-Marie Määttä, Raimo A. Ketola, Marjo Yliperttula, Thomas Wirth, Arto Urtti

**Affiliations:** 1 Centre for Drug Research, Faculty of Pharmacy, University of Helsinki, Helsinki, Finland; 2 Division of Biopharmacy and Pharmacokinetics, Faculty of Pharmacy, University of Helsinki, Helsinki, Finland; 3 Ark Therapeutics, Kuopio, Finland; 4 Department of Biotechnology and Molecular Medicine, University of Eastern Finland, Kuopio, Finland; 5 Department of Biosciences, University of Eastern Finland, Kuopio, Finland; National Cancer Center, Japan

## Abstract

**Background:**

Epidermal growth factor receptor (EGFR) is overexpressed in many solid tumor types, such as ovarian carcinoma. Immunoliposome based drug targeting has shown promising results in drug delivery to the tumors. However, the ratio of tumor-to-normal tissue concentrations should be increased to minimize the adverse effects of cytostatic drugs.

**Methodology/Principal Findings:**

We studied the EGFR-targeted doxorubicin immunoliposomes using pre-targeting and local intraperitoneal (i.p.) administration of the liposomes. This approach was used to increase drug delivery to tumors as compared to direct intravenous (i.v.) administration of liposomes. EGFR antibodies were attached on the surface of PEG coated liposomes using biotin-neutravidin binding. Receptor mediated cellular uptake and cytotoxic efficacy of EGFR-targeted liposomes were investigated in human ovarian adenocarcinoma (SKOV-3 and SKOV3.ip1) cells. *In vivo* distribution of the liposomes in mice was explored using direct and pre-targeting approaches and SPECT/CT imaging. Targeted liposomes showed efficient and specific receptor-mediated binding to ovarian carcinoma cells *in vitro*, but the difference in cytotoxicity between targeted and non-targeted liposomes remained small. The relatively low cytotoxic efficacy is probably due to insufficient doxorubicin release from the liposomes rather than lack of target binding. Tumor uptake of targeted liposomes *in vivo* was comparable to that of non-targeted liposomes after both direct and pre-targeting administration. For both EGFR-targeted and non-targeted liposomes, the i.p. administration increased liposome accumulation to the tumors compared to i.v. injections.

**Conclusions/Significance:**

Intraperitoneal administration of liposomes may be a beneficial approach to treat the tumors in the abdominal cavity. The i.p. pre-targeting method warrants further studies as a potential approach in cancer therapy.

## Introduction

In normal conditions, the epidermal growth factor receptor (EGFR) is involved in cell growth, differentiation and repair. Many solid tumor types, e.g., breast, colon, pancreatic, lung, and ovarian cancers, overexpress EGFR, thereby leading to tumor progression, invasion, and metastases [1], [2]. Therefore, EGFR is a potential target in cancer treatment.

Specific drug targeting to tumors is a challenging task. Liposomal drug formulations have shown improved doxorubicin delivery to the tumors [3]. Liposomes with polyethylene glycol (PEG) based steric stabilization circulate over prolonged periods in blood stream and slowly accumulate into tumors. Blood vessels in tumors have 100–600 nm gaps between the endothelial cells, whereas the endothelia in healthy blood vessels are continuous [4]. Passive accumulation of long-circulating PEG coated liposomes (size 100–200 nm) is based on enhanced permeation and retention (EPR) effect [5]. Active targeting of liposomes to the cancer cells is based on liposome functionalization with targeting moieties. Targeting can be accomplished with direct targeting or pre-targeting methods. In direct targeting, the targeting antibodies are coupled to the liposomal surface. The resulting immunoliposomes are administered as such. Immunoliposomes show cellular targeting *in vitro*, but *in vivo* there are still many drug delivery hurdles. These issues include liposome stability in blood circulation, their sequestration from the blood stream by reticulo-endothelial system (RES), immunogenicity, penetration into the solid tumors, specific uptake to the tumor cells, and drug release at the target site [6].

Pre-targeting technology has been developed to minimize the exposure of patients to radioactive compounds that are used in cancer imaging and radioimmunotherapy [7]–[9]. The target-specific antibody is injected first and, thereafter, radiolabeled small molecule is administered. The radioligand should bind to the pre-localized antibody in the target tissue, but the unbound radioligand is eliminated rapidly renally [10]. Pre-targeting is based on high affinity biotin-avidin coupling (K_d_ ∼ 10^15^ M^−1^) [11], [12] or bispecific antibodies [8]. Pre-targeting has been utilized in targeting of polymeric nanoparticles [13], [14] and liposomes [15], [16] to cancer cells *in vitro*.

Some cancers, including ovarian cancer, frequently spread to the peritoneal cavity. Therefore, intraperitoneal (i.p.) administration might be beneficial in targeting both primary tumor and peritoneal metastases. It was recently shown that PEGylated liposomes accumulate in tumors and ascites after i.p. injection [17], [18]. Zavaleta *et al.* used avidin to aggregate biotin-liposomes in the abdominal cavity to prolong drug retention in peritoneum and associated lymph nodes where metastatic cancer cells may be located [19].

In our study, we combined pre-targeting and local i.p. application of liposomes to improve tumor targeting of doxorubicin beyond the levels achievable with direct intravenous targeting. We studied *in vitro* uptake and functionality of EGFR-targeted liposomes in human ovarian adenocarcinoma (SKOV-3 and SKOV3.ip1) cells. Thereafter, *in vivo* distribution and tumor accumulation of liposomes was studied in mice using both direct and pre-targeting approaches. EGFR-binding antibody cetuximab (Erbitux®) was linked to the liposomes via biotin-neutravidin binding. Our results suggest that pre-targeting and i.p. administration of liposomal drugs may be feasible drug delivery approach in the treatment of ovarian tumors.

## Results

### Targeting of Liposomes in SKOV-3 and CV-1 Cells

**Figure 1:**
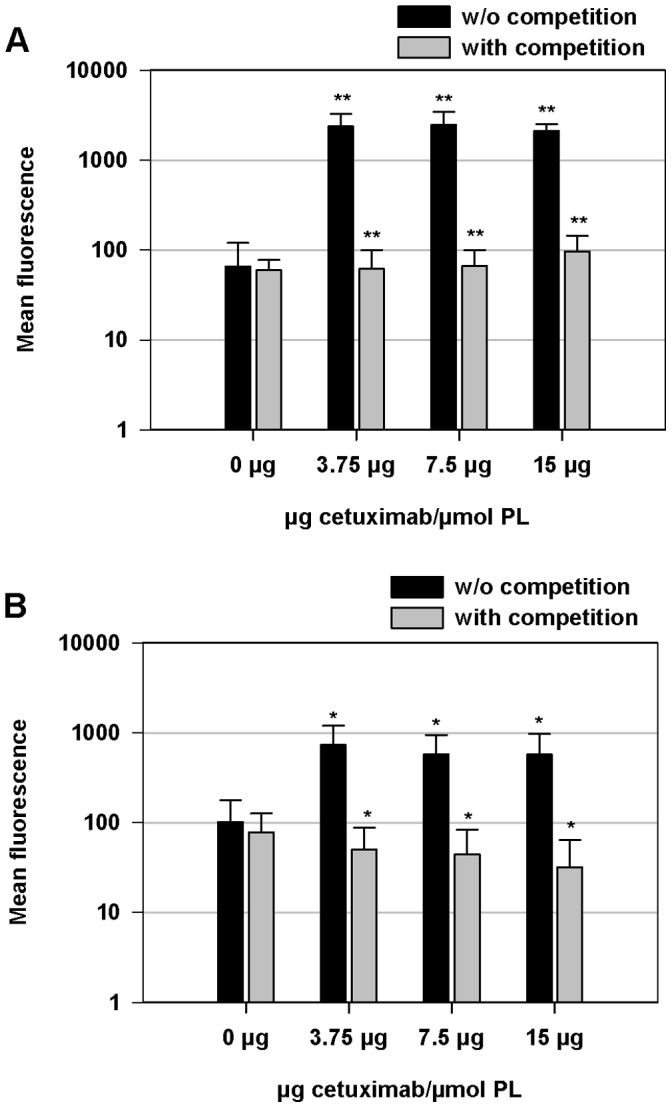
Cellular uptake of non-targeted and EGFR-targeted fluorescein-labeled liposomes. The SKOV-3 (A) or CV-1 (B) cells were either treated with or without competition with free cetuximab for 1 h at 4°C, washed and incubated with biotin-liposomes (0 µg cetuximab/µmol PL) or cetuximab-biotin-liposomes (3.75; 7.5 or 15 µg cetuximab/µmol PL) for 2 h at 37°C. After incubation, the cells were washed, detached and analyzed by flow cytometry for liposome uptake. The data indicates mean fluorescence ± SD. *p*<0.005 (*), *p*<0.001 (**).

Cetuximab-biotin-liposomes were taken up efficiently by SKOV-3 cells. In the competition study, free cetuximab decreased the uptake of the cetuximab-biotin-liposomes to the level of non-targeted biotin-liposomes (Fig. 1A). In the presence of free cetuximab the fluorescence levels decreased to 22–38 times lower levels as compared to the situation without free cetuximab competition (*p*<0.001). The same trend was seen in monkey kidney fibroblast (CV-1) cells, even though the specific uptake in these cells was lower, being 13–17 times higher than in the presence of the antibody competition (*p*<0.005) (Fig. 1B). Based on our results, the antibody density of 7.5 µg mAb/µmol phospholipid (5 mAb molecules/liposome) was adequate to target the liposomes to SKOV-3 cells (Fig. 1A). Further increase of mAb concentration did not enhance the cellular uptake.

### Cytotoxicity of Doxorubicin Liposomes in SKOV-3 and CV-1 Cells

**Figure 2:**
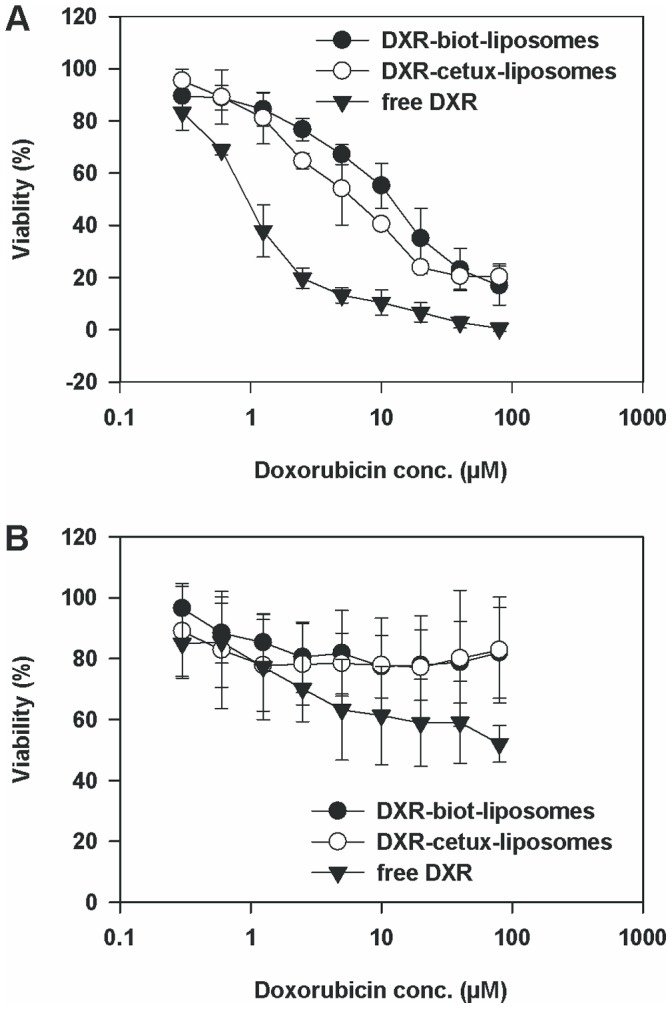
Cytotoxicity of EGFR-targeted and non-targeted doxorubicin-liposomes and free doxorubicin. SKOV-3 (A) and CV-1 (B) cells were exposed to liposomal and free doxorubicin (DXR) (0.3–80 µM) for 2 h. After exposure to the drug, the cells were washed and incubated in growth medium for 5 days. Cell growth was assayed using Alamar Blue®. The data are presented as mean ± SD.

Cytotoxic activity of doxorubicin-cetuximab-biotin-liposomes was compared to doxorubicin-biotin-liposomes and free doxorubicin in SKOV-3 and CV-1 cells. After 2 h liposome exposure and further incubation for 5 days, increased toxicity of doxorubicin-cetuximab-biotin-liposomes (IC_50_ = 5.5±1.5 µM) was seen in SKOV-3 cells compared to the doxorubicin-biotin-liposomes (IC_50_ = 11.8±4.4 µM) (Fig. 2A). At doxorubicin concentration of 10 µM, the cytotoxic efficacy of doxorubicin-cetuximab-biotin-liposomes was higher than the efficacy of non-targeted biotin-liposomes (*p*<0.005). Both doxorubicin liposome types were less toxic than free doxorubicin (IC_50_ = 0.9±0.2 µM). Further incubation of the cells (for 7 days) did not affect toxicity of the liposomal or free doxorubicin (data not shown). In CV-1 cells both liposome formulations demonstrated only marginal toxicity after 5 days, whereas free doxorubicin showed higher cellular toxicity (Fig. 2B). However, IC_50_ was >80 µM in all cases.

### Distribution of Doxorubicin Liposomes in SKOV-3 Xenograft Bearing Mice

**Figure 3:**
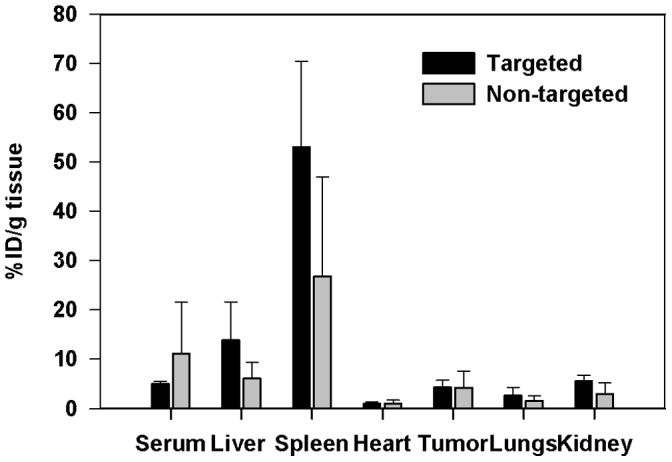
Biodistribution of EGFR-targeted and non-targeted doxorubicin-liposomes in mice bearing i.p. SKOV-3 xenografts. Either targeted liposomal DXR or non-targeted liposomal DXR was injected i.v. at a dose of 2 mg/kg. DXR content was assayed in the indicated tissues at 24 h post-treatment (*n* = 3). Data are expressed as mean of % of injected dose (ID)/g tissue, ± SD.

Distribution and tumor accumulation of doxorubicin-cetuximab-biotin-liposomes and doxorubicin-biotin-liposomes was investigated after the injection of the liposomes i.v. to the mice bearing i.p. SKOV-3 tumors. The main organs and the tumors were dissected 24 h post-injection and the concentration of doxorubicin in the tissue samples was analyzed by liquid chromatography-mass spectrometry (LC-MS). Drug levels in spleen, liver, and kidneys were approximately two times higher in doxorubicin-cetuximab-biotin-liposomes treated mice than in the doxorubicin-biotin-liposome group (Fig. 3). On the contrary, drug concentrations in the serum of doxorubicin-cetuximab-biotin-liposome treated mice were about a half of the concentrations in doxorubicin-biotin-liposome treated animals. Accumulation of doxorubicin to the other organs, such as heart, lungs, and brain, was minimal. Uptake in the tumor tissue was ∼5% of the injected dose (ID)/g tissue for both formulations.

### Pre-targeting Approach: *in vitro* Targeting Efficacy

**Figure 4:**
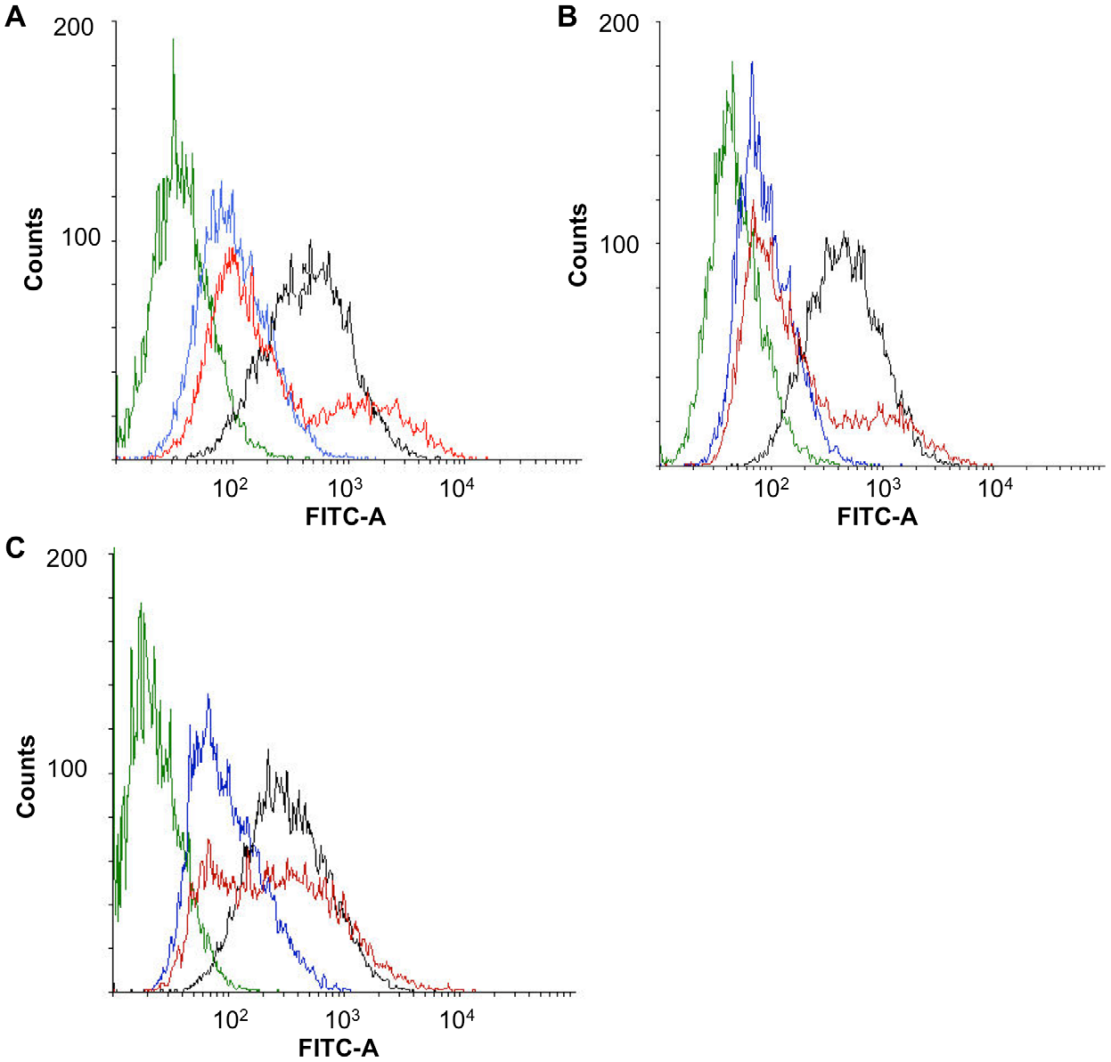
Flow cytometric analysis of cellular affinity. Directly targeted (black), pre-targeted (red) and non-targeted (blue) fluorescein-labeled liposomes were incubated with SKOV-3 (A) and SKOV3.ip1 (B–C) cells. In the pre-targeting group, the cells were incubated with neutravidin-cetuximab for 4 h, washed and incubated with biotin-liposomes for 2 h (A–B) or 4 h (C). In the other groups, the cells were incubated with the liposomes for 2 h (A–B) or 4 h (C). The green line is representing the background fluorescence of untreated cells.

The pre-targeting method was tested *in vitro* in SKOV-3 and SKOV3.ip1 cells. The cells were first incubated with neutravidin-cetuximab for 4 h, washed, and then incubated with biotin-liposomes for 2 h. Results in Fig. 4A–B show biphasic cell uptake profiles of liposomes in both cell lines after pre-targeting. The higher peak of the profile is under the uptake curve of non-targeted liposomes and the lower peak is under the uptake curve of directly targeted liposomes. However, the mean fluorescence of the liposomes bound to the cells is at the same level after pre-targeting and direct targeting (Fig. S1). The broad distribution of the fluorescence values after pre-targeting is not taken into account in the mean values and, therefore, such values may be misleading. Shorter incubation time with neutravidin-cetuximab (30 min, 1 or 2 h), lowered the level of association of the pre-targeted liposomes with the cells (data not shown). Whereas longer incubation time of 4 h of pre-targeted biotin-liposomes resulted in higher levels of active targeting to the cells (Fig. 4C).

### SPECT/CT Imaging and *in vivo* Distribution of Pre-targeted Liposomes

**Figure 5:**
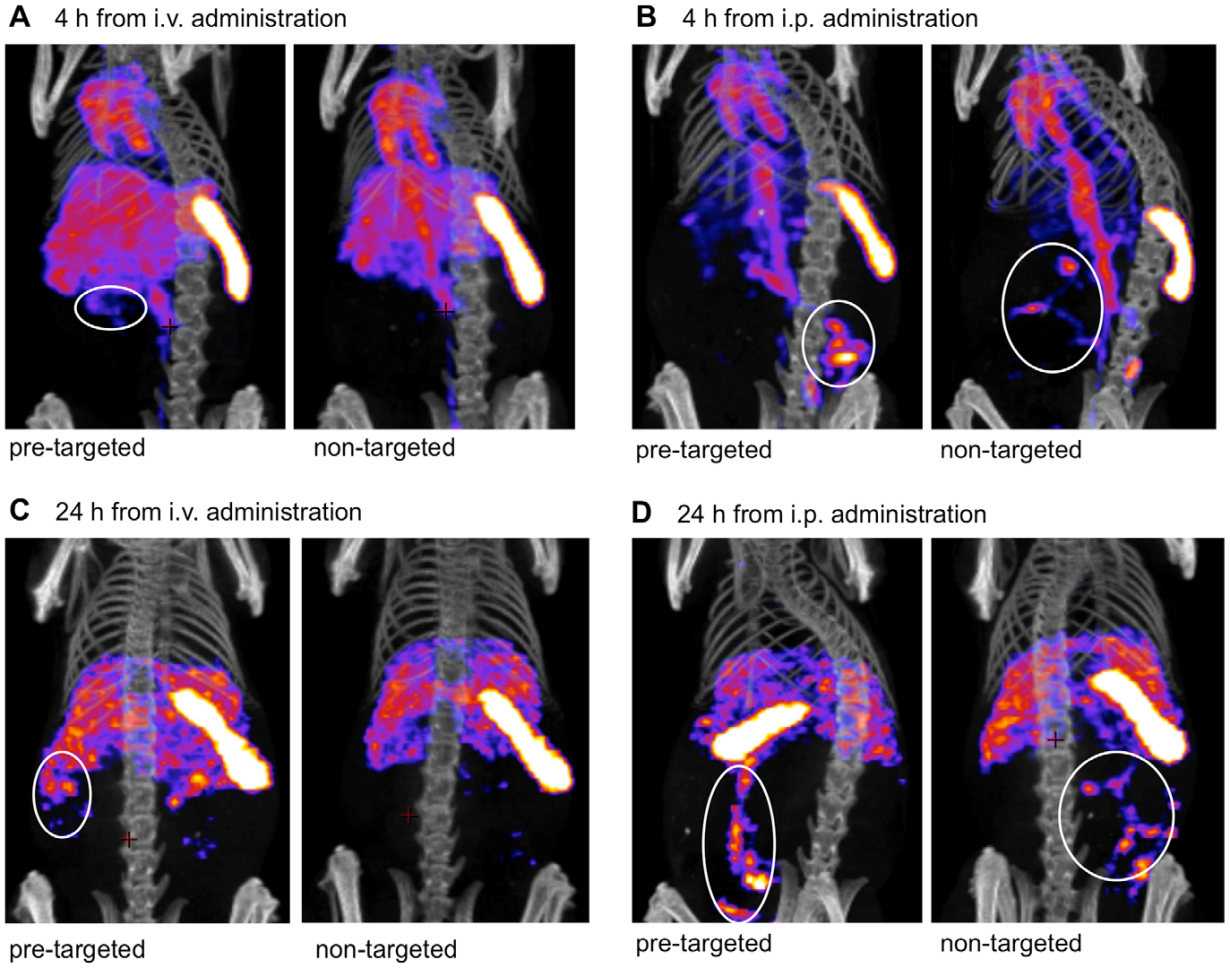
SPECT-CT imaging. SPECT-CT images of pre-targeted and non-targeted ^99m^Tc-liposomes 4 h (A–B) and 24 h (C–D) after injection of liposomes, administrated either i.v. (A, C ) or i.p. (B, D). Neutravidin-cetuximab was injected to pre-targeted groups and PBS to non-targeted groups i.p. 24 h before liposome injections. Tumors are marked with white circles on the figures. Minimum and maximum values of intensity were adjusted to the same scale for 4 h images and for 24 h images, respectively.

Pre-targeting approach in liposomal drug delivery was studied using mice bearing i.p. SKOV3.ip1 tumors. The mice received first i.p. injections of neutravidin-cetuximab or phosphate buffered saline (PBS, control). Then, 24 h later, ^99m^Tc-labeled biotin-liposomes were given i.v. or i.p. Single photon emission computed tomography/computer tomography (SPECT/CT) images revealed that 4 h post-injection most i.v. administered liposomes were in blood circulation, spleen and liver (Fig. 5A). At 4 h after i.p. injection, the liposomes had been cleared from the abdominal cavity to blood circulation and part of the liposomes distributed to the spleen and tumor (Fig. 5B). At 24 h post-injection (i.v. and i.p.) the liposomes localized in spleen, liver and tumor (Fig. 5C–D).

**Figure 6:**
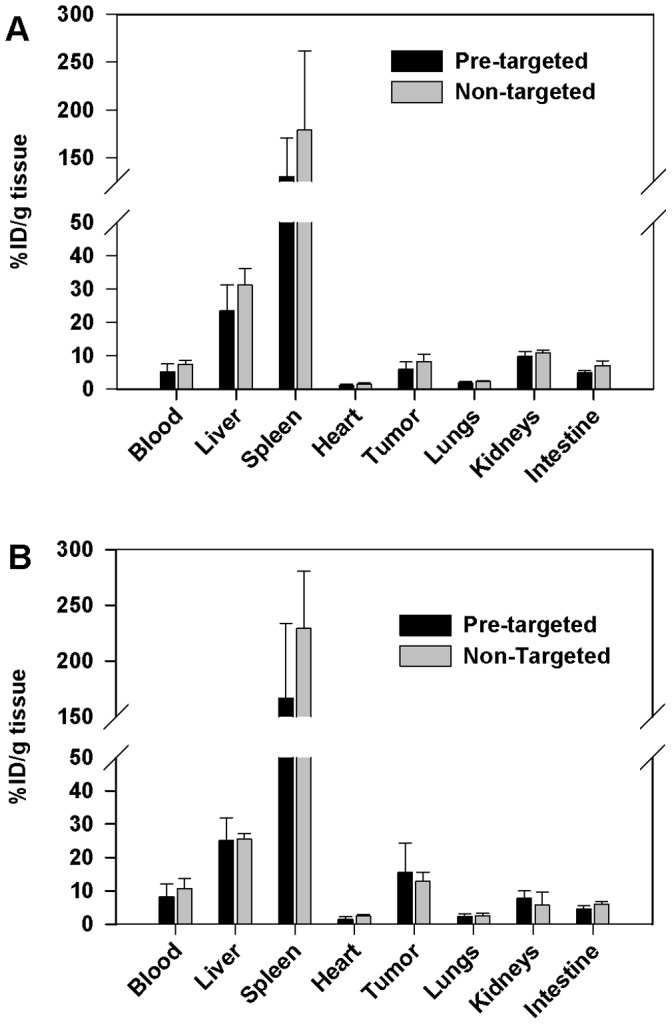
Biodistribution of pre-targeted and non-targeted liposomes in mice bearing i.p. SKOV3.ip1 xenografts. Either neutravidin-cetuximab, at a dose of 20 µg of antibody, or PBS was injected i.p. to the mice. ^99m^Tc-labeled biotin-liposomes were injected 24 h later i.v. (A) or i.p. (B). Radioactivity of the indicated tissues was determined 24 h from liposome injections. The results are expressed as %ID/g tissue ± SD for pre-targeted liposomes and for non-targeted liposomes (*n* = 3–4).

Post-mortem evaluation of distribution in the dissected organs after 24 h from liposome injections shows that the highest activity was in spleen after both administration ways (Fig. 6A–B). Activity was found also in the liver, tumor, blood, kidneys, and intestine, but less in the lungs and heart. The two routes of administration (i.v. and i.p.), resulted mostly in similar distribution, even though the i.p. injection resulted in higher average liposome concentration in blood and tumors than i.v. injection. The differences in tumor were 2.6 fold for pre-targeted systems (*p* = 0.083) and 1.6 fold for non-targeted liposomes (*p* = 0.053). Pre-targeted, i.p. injected liposomes resulted also in higher tumor-to-blood ratio (2.2±1.7) compared to pre-targeted, i.v. injected liposomes (1.3±0.5).

## Discussion

In this study, we evaluated the targeting efficiency of anti-EGFR-liposomes to ovarian carcinoma. Biotin-neutravidin technology in antibody coupling was used because it is suitable for both direct and pre-targeting approaches. Since ovarian tumors often spread to the peritoneal cavity, i.p. route of liposome administration was compared i.v. delivery.

First, we studied direct targeting of immunoliposomes with cetuximab coupled to the PEGylated liposomes via biotin-neutravidin link. According to the literature [20], [21], SKOV-3 cells have high EGFR-expression level that makes it a suitable cell model for studies of EGFR-targeted liposomes. We demonstrated high and specific binding of the cetuximab-biotin-liposomes to SKOV-3 cells in a cellular competition study. Some EGF-receptor specific binding could be seen also in non-malignant control cells (CV-1). To minimize the immunological effects, the lowest required number of antibodies per liposome was evaluated. This approach should minimize antibody mediated clearance of liposomes from the blood circulation [22]. Based on our results, five antibody molecules/liposome was sufficient to achieve efficient targeting to SKOV-3 cells. This antibody density was used in further experiments.

Cetuximab-biotin-liposomes were more toxic to SKOV-3 cells than non-targeted biotin-liposomes at DXR concentration of 10 µM. Even though the difference was statistically significant, it was modest. However, Figure 1A shows 22–38 times higher binding of the targeted liposomes over the non-targeted ones on the SKOV-3 cells. Based on these results the relatively small difference in cytotoxicities of targeted and non-targeted liposomes may be due to insufficient doxorubicin release from the liposomes rather differences in the cellular access. Poor release of doxorubicin from the liposomes may also explain higher cell killing activity of free drug compared to targeted liposomes. Drug release from liposomes may be triggered using pH sensitive [23], [24] or thermosensitive liposomal formulations [25], [26]. For example, Kirchmeier *et al*. showed 12 to 35 times higher nuclear accumulation of doxorubicin after exposing the Namalwa cells to targeted pH-sensitive liposomes (dioleylphosphatidylethanolamine (DOPE)/cholesteryl hemisuccinate (CHEMS)/DSPE-PEG_2000_ or DOPE/DSPE-PEG_2000_) instead of targeted HSPC/Chol/DSPE-PEG_2000_ liposomes [27]. It should be noted, that our results do not reflect cetuximab effects as such, since the IC_50_ of cetuximab alone is 1700 µM in the SKOV-3 cells [28], a concentration of four orders of magnitude higher than the highest cetuximab concentration used in our *in vitro* toxicity study.

Distribution study *in vivo* indicated more rapid clearance of directly targeted doxorubicin-cetuximab-biotin-liposomes compared to non-targeted liposomes even if the amount of antibodies was decreased to five molecules/liposome. Apparently, minimizing the antibody density on the liposomal surface does not avoid the immunological defense surveillance. Even though the targeted liposomes were more efficiently removed by the RES, their tumor accumulation was comparable to the non-targeted liposomes. By using smaller fractions of antibodies, Fab-fragments or single chain Fv-antibody fragments (scFv), the blood clearance may be decreased to the levels of PEGylated liposomes [5]. In addition to the form of the conjugated antibody, also the linking system (biotin-neutravidin-biotin) probably contributes to the clearance of the targeted liposomes.

Instead of using smaller antibody fragments to prepare immunoliposomes we chose another approach. We utilized pre-targeting technology based on the hypothesis that compared to direct injection of immunoliposomes the separate injections of antibodies and liposomes would result in prolonged half-life and diminished RES mediated removal. Potentiated immunogenicity has been shown when cetuximab was coupled to the PEGylated liposomes, whereas separate injections of PEGylated liposomes and cetuximab or even co-injection of uncoupled liposomes and cetuximab did not cause immunological reactions [22]. In pre-targeting, the tumor-homing antibody is administered and allowed to accumulate into the tumor. Thereafter, drug-loaded liposomes are administered. In our case, the antibody was linked to neutravidin, which is chemically deglycosylated avidin with reduced non-specific binding to cells and increased circulation time compared to avidin. Yet, neutravidin and avidin have equal biotin binding affinities. Optimally, tumor attached neutravidin-cetuximab binds to biotinylated liposomes and the complex then enters to the target cell. In addition to possibly reduced immunological reactions, this method may enable simultaneous targeting to different antigens with the same biotinylated liposomes.

Both ovarian carcinoma cell lines, SKOV-3 and SKOV3.ip1, showed biphasic binding of the pre-targeted liposomes indicating that pre-targeting results in liposome binding both in EGFR-specific and non-specific manner. Thus, it is possible that some neutravidin-cetuximab was already internalized to the cells before its binding with biotin-liposomes. Using slowly internalizing anti-HER2 mAbs, pre-targeting of poly-(lactic acid) PLA nanoparticles was shown to be as efficient as direct targeting in SKOV-3 cells [13]. In our study, shorter incubation time with neutravidin-cetuximab (30 min, 1 or 2 h), unlike expected, did not increase the level of association of the liposomes to the cells. Hence, it seems that biotin-liposomes would benefit from the longer incubation time (2 h vs. 4 h) thus giving more time for binding of liposomal biotin with neutravidin on cetuximab. Biotin is a small molecule (244 g/mol), which could be partly covered by PEG shield of the liposome surface. Interference of the PEG coating was also noticed with folic acid (FA) target binding, especially when FA was linked to the PEG arm having the same length as the PEG coating on the liposome surface [29]. That could be due to the low molecular weight of FA (441 g/mol) but also to its potential to form hydrogen bonds with PEG. Recently, peptide ligand was shown to interfere with PEG chains on the liposomal surface, thereby reducing the targeting functionality [30].

For the *in vivo* pre-targeting experiments, we chose the SKOV3.ip1 cell line that grows more aggressively and homogenously tumors compared to SKOV-3 cells [31]. As a first step, NA-cetuximab or phosphate buffered saline (PBS, control) was injected into the peritoneal cavity of the mice. Previously, no difference of cetuximab distribution was seen after i.p. and i.v. injections [32]. As a second step, ^99m^Tc-labeled biotin-liposomes were given either i.v. or i.p. 24 h later, sufficient time for cetuximab accumulation to SKOV-3 tumors [33].

SPECT-CT analyses showed presence of i.v. delivered liposomes in blood circulation, spleen, and liver at 4 h post-injection (Fig. 5A). At 4 h the i.p. liposomes had been eliminated from the abdominal cavity to blood circulation and started to accumulate in the spleen and the tumor (Fig. 5B). This is consistent with the study of Zavaleta *et al*. who used i.p. injected biotin-liposomes and saw liposomal accumulation in the tumor at 4 h after i.p. delivery [19]. In fact, liposomal delivery to the tumors may be faster after i.p. than i.v. administration, because the tumor is in the peritoneal cavity. Compared to i.v. liposomes, Lin *et al*. found more rapid tumor accumulation of i.p. administered and ^111^In labeled liposomes [18]. Like in our study, the tumor levels of i.p. injected liposomes remained high at 24 h post-injection. Prolonged blood circulation of liposomes after i.p. administration may result from the slow liposome absorption from the peritoneal cavity [34]. At 24 h post-injection, most pre-targeted liposomes were found in spleen. High splenic uptake is often related to large liposome size (e.g. due to aggregation), but in this study no aggregation was seen and both non-targeted and targeted liposome showed splenic deposition.

Accumulation of liposomes in the tumor was higher after i.p. injection than after i.v. injection at 24 h time point for both pre-targeted (2.6-fold higher) and non-targeted (1.6-fold higher) liposomes. Pre-targeted, i.p. injected liposomes resulted also in higher tumor-to-blood ratio (2.2±1.7) than i.v. injected liposomes (1.3±0.5). These results suggest that pre-targeting and i.p. administration of liposomal drugs could be applicable in drug delivery to ovarian tumors. Unfortunately, the small animal groups in each study (*n* = 3–4), does not enable statistical evaluation of the results.

Pre-targeting approach did not increase the tracer levels in the tumor tissue significantly *in vivo* over non-targeted liposomes even though cell specific targeting was observed *in vitro*. Based on earlier studies [35], [36], accumulation of liposomes in the tumor does not necessarily reflect its therapeutic potential. Mamot *et al.* showed that even if the anti-EGFR immunoliposomes did not increase tumor accumulation over non-targeted liposomes, the immunoliposomes showed improved internalization into the target cells and enhanced the therapeutic efficacy [35]. Similar conclusion was also presented by Kirpotin *et al*., who found that anti-HER2 immunoliposomes did not increase uptake in the tumor tissue compared to non-targeted liposomes, but the intracellular uptake in cancer cells was enhanced up to 6-fold due to targeting [36]. In the light of these previous studies enhanced therapeutic efficacy might be achieved also with our pre-targeted liposomes even if the tumoral accumulation was not improved compared to the non-targeted liposomes. The rate-limiting step for tumor localization of liposomes, targeted or not, is extravasation from tumor vasculature that is required for specific cell internalization of liposomes.

For further development of our pre-targeting method, there are some issues that need to be considered. Cellular internalization of pre-targeted cetuximab can compromise specific tumor targeting of the liposomes. The antibodies should remain accessible at the target site to allow specific liposome binding. Too rapid cellular internalization of the antibodies would hamper targeted liposomal delivery. On the other hand, internalization of the therapeutic liposomes is requirement of improved therapeutic efficacy [35], [37]. Slowly internalizing antibodies are potentially useful technology in this context. Pre-targeting approach requires optimized timing between the injections of neutravidin-cetuximab and biotin-liposomes. Half-life of cetuximab is 2.9 days in bloodstream in mice [38], but the influence of neutravidin on the circulation time, immunogenicity and tumor accumulation should be evaluated.

In summary, direct targeting of EGFR-targeted liposomes was specific and efficient in ovarian cancer cells *in vitro*, but tumor accumulation *in vivo* was comparable to that of non-targeted liposomes. However, tumor accumulation of i.p. liposomes was faster and reached higher levels than i.v. liposomes. It may be beneficial to use i.p. delivery for treatment of abdominal tumors. Both local and systemic drug delivery to the peritoneal tumors might be achieved with i.p. pretargeting method. In this case, rapid drug delivery to the tumors would be accomplished, followed by systemic delivery from the bloodstream.

## Materials and Methods

### Biotinylation of Cetuximab

The glycine buffer of cetuximab (Erbitux®, Merck) was changed to PBS (pH 7.4) with a centrifugal concentrator tube (Vivaspin 20, 10 K MWCO). Three ml of a 5 mg/ml solution of cetuximab in PBS was mixed with 250 µl of a 10 mM solution of N-hydroxysuccinimidobiotin (NHS-biotin, Pierce, Rockford, USA) in dimethylsulfoxide. The reaction was left for 30 min at room temperature. The biotinylated cetuximab was purified by centrifugal concentrator (Vivaspin 20, 10 K MWCO) to remove the free biotin molecules and prepared in PBS at 5.0 mg/ml.

Avidin conjugated horseradish peroxidase (HRP) enzyme immunoassay (EIA) was used to detect biotin on cetuximab surface. Goat anti-human IgG HRP EIA was used to verify usability and to determine an appropriate concentration of cetuximab-biotin. Cetuximab and 10 mM NaHCO_3_ were used as controls. Cetuximab-biotin and cetuximab were serially diluted (20 µg/ml to 0.3125 µg/ml) in 10 mM NaHCO_3_ (pH 9.5), placed in duplicates 100 µl/well into 96-well plate (Enhanced Binding (EB) Combiplate, Thermo Labsystems Oy, Finland) and incubated at room temperature overnight. The antibody-coated wells were washed three times with PBST (0.05% Tween-20 in PBS) to remove excess antibodies and were then blocked 100 µl/well with 1% bovine serum albumin (BSA; Rockland, Gilbertsville, USA) in PBST. After 1 h incubation at room temperature the wells were washed three times with PBST. Goat anti-human IgG HRP conjugate or avidin-HRP conjugate (both diluted 1∶10 000 in 1% BSA in PBST) (Rockland, Gilbertsville, USA) 100 µl/well was added and incubated for 30 min at room temperature. After three washes with PBST, 100 µl/well of 5′5′, 3′3′tetramethylbenzidine substrate solution was added and kept in the dark for 10 min. The reaction was stopped by adding 50 µl of 2 M H_2_SO_4_. The optical density was determined at 450 nm (OD_450_) using an ELISA reader (Labsystems Multiscan RC, Thermo Labsystems Oy, Finland).

### Liposome Preparation

Liposomes were composed of fully hydrogenated soy phosphatidyl choline (HSPC), cholesterol (Chol), distearoylphosphatidylethanolamine-polyethylene glycol-2000 (DSPE-PEG_2000_) and DSPE-PEG_2000_-biotin, 2∶1:0.08:0.02 (mol:mol) with 0.2% of fluorescein-phosphatidylethanolamine (Fluor-PE). All lipids were from Avanti Polar Lipids (Alabaster, Alabama, USA). Chloroform solutions of the lipids were mixed and chloroform was evaporated with rotary evaporation. Formed thin lipid film was hydrated with PBS (pH 7.4) in a 65°C water bath for 30 min following five cycles of freezing and thawing. The liposomes were sized by repeated extrusion (LIPEX Extruder, Northern Lipids Inc, Canada) at 65°C through a polycarbonate membrane with a pore size of 100 nm.

For encapsulation of doxorubicin, the remote-loading method [39] was used. First, the lipid film was hydrated in 250 mM ammonium sulfate (pH 5.5). After extrusion, the outer buffer of the liposomes was changed to 100 mM acetate/70 mM NaCl, pH 5.5 in a Sephadex G-50 column (Sigma-Aldrich). Doxorubicin (DXR, Sigma-Aldrich) was encapsulated into the liposomes at DXR:HSPC, 0.2∶1 (w:w) during incubation at 65°C for 30 min. Free DXR was removed in Sephadex G-50 column equilibrated with 20 mM HEPES/150 mM NaCl (pH 7.4).

Neutravidin (Invitrogen) and biotinylated cetuximab were coupled in 1∶1 molar ratio at 40°C for 1 h. Formed complex was integrated onto the biotinylated liposomes (biotin-liposomes) of varying cetuximab:phospholipid (PL) ratios (7.5, 15 and 30 µg of cetuximab/µmol PL) at 40°C for 1 h. The mixture was passed through a Sepharose CL-4B column (Sigma-Aldrich) to remove unbound cetuximab. As a result, cetuximab-biotin-liposomes were formed.

### Characterization of Liposomes

The particle size was measured by Zeta-Sizer (3000 HS, Malvern Instruments Ltd, UK). Size of the liposomes varied from 100 nm to 130 nm. Phospholipid (PL) concentration was determined by fluorimetry using fluor-PE as a probe (λ_ex_ = 497 and λ_em_ = 521 nm). The amount of DXR encapsulated inside the liposomes was determined from its absorbance at 492 nm. Encapsulation efficacy was always more than 90%. Cetuximab coupling efficiency was evaluated using fluorimetry at wavelengths of λ_ex_ = 285 and λ_em_ = 335, being ∼50%.

### Cell Lines

SKOV-3, EGFR positive human ovarian adenocarcinoma cell line was received from Ark Therapeutics, Kuopio, Finland (originally from American Type Culture Collection (ATCC)). Cells were cultured in McCoy’s 5A medium with Glutamax (Gibco) supplemented with 10% fetal bovine serum (FBS, Gibco) and with 1% penicillin/streptomycin (P/S, Gibco). SKOV3.ip1, established by Yu *et al.* [31] was a kind gift from Prof. A. Hemminki (Cancer Gene Therapy Group, Biomedicum, Helsinki, Finland). SKOV3.ip1 cell line is a derivative of SKOV-3 cell line showing more aggressive and homogenous tumor growth pattern compared to parental cell line. The cells were cultured in Dulbecco’s modified Eagle Medium (DMEM, 31885, Gibco), supplemented with 10% FBS and 1% P/S. Monkey kidney fibroblast cell line, CV-1, was purchased from ATCC and used as a control. CV-1 was cultured in DMEM (31885) supplemented with 10% FBS and 1% P/S. All cell lines were grown at 37°C, in 5% CO_2_ and sub-cultured twice a week.

### Cellular Uptake/Affinity Studies after Direct and Pre-targeting

Receptor mediated cellular uptake was determined using flow cytometry. One day before experiment, SKOV-3 and CV-1 cells were seeded on 6-well plates, 2×10^5^ and 1×10^5^ cells/well, respectively. In a competition study, the cells were incubated with free cetuximab (8 µg/well) or with growth medium for 1 h at 4°C. After incubation, the cells were washed with PBS and cetuximab-biotin-liposomes and biotin-liposomes were added at a concentration of 0.1 mM of PL for 2 h, at 37°C. Cells were washed twice with PBS, then with 1 M sodium chloride, again with PBS, and detached from the wells with 0.25% trypsin/0.2 M EDTA. Then the cells were fixed in 1% paraformaldehyde (PFA) solution for 10 min, centrifuged (6 000 rpm, 10 min) and washed once more with PFA, centrifuged and suspended in 0.5 ml of PFA. Analysis was carried out with fluorescence activated cell sorting (FACS) (BD LSRII, BD Biosciences, USA) using specific wavelengths for fluorescein (λ_ex_ = 497 and λ_em_ = 521 nm).

Cellular affinity after pre-targeting was studied in SKOV-3 and SKOV3.ip1 cells, seeded onto 6-well plates, both at the density of 2×10^5^cells/well. On the next day, the cells were pre-targeted with cetuximab:neutravidin, 1∶1 (mol/mol), at a dose of 8 µg of cetuximab/well in biotin-free growth medium (DMEM 31885) for 30 min, 1, 2 or 4 h at +37°C. Growth medium was given as a control. Next, the cells were washed with PBS and biotin-liposomes were added and incubation was continued for 2 or 4 h at +37°C under gentle shaking. After incubation the cells were washed twice with PBS and prepared for FACS as described previously.

### Cytotoxicity Studies

The cytotoxic efficacy of DXR-loaded cetuximab-biotin-liposomes, biotin-liposomes and free DXR was compared. SKOV-3 and CV-1 cells were seeded on 96-well plates at a density of 6 500 and 5 000 cells/well, respectively. On the next day, cetuximab-biotin-liposomes, biotin-liposomes and free DXR were added to the cells using DXR concentration series of 0, 0.3, 0.6, 1.25, 2.5, 5, 10, 20, 40 and 80 µM for 2 h at +37°C. Then, the cells were washed twice with PBS and incubated in fresh growth medium. Alamar Blue™ assay was done 5 and 7 days after the treatment. The cells were stained with 10% Alamar Blue™ and fluorescence was measured at wavelengths of λ_ex_ = 530 and λ_em_ = 590 nm. The percentage of viability was calculated by comparing treated cells with untreated cells that represent 100% viability.

### Biodistribution of DXR-loaded Immunoliposomes

This animal study was approved by Provincial Government of Southern Finland (ESLH-2008-03724/Ym-23) and performed in accordance with Good Laboratory Practices for Animal Research. Eight BALB/ca female nude mice (Harlan, Netherlands) were injected i.p. with 1×10^6^ SKOV-3 cells to establish an ovarian cancer model. On day 17 after tumor cell injection, DXR-containing cetuximab-biotin-liposomes and biotin-liposomes were injected i.v. with a DXR dose of 2 mg/kg. The mice were sacrificed 24 h after liposome administration by CO_2_ and cervical dislocation. Blood samples were taken by cardiac puncture and serum was separated. Tissue samples were collected and frozen in liquid nitrogen. All samples were kept frozen (−70°C) until DXR extraction.

### Extraction of DXR from Tissues for LC-MS Analysis

The procedure for extraction of DXR was modified from the ones described by Jong et al. and Hsieh et al. [40], [41]. The tissue samples (∼100 mg of each) were cut into small pieces, and 50 µl of 0.6 µM daunorubicin (DNR, Sigma-Aldrich), the internal standard (ISTD), was added to all samples. To extract DXR from the tissues, 200 µl of 5% silver nitrate, 200 µl of 100 mM ammonium formate and 400 µl of acetonitrile (ACN) (all from Sigma-Aldrich) were added and mixture was vortexed. The samples were homogenized with a probe sonicator (Branson Sonifier 450, USA), centrifuged for 10 min at 3220×g, at +4°C and the supernatant was taken. The remaining pellet was treated once again in aforementioned way. The supernatants of both treatments were mixed and the solvent was evaporated in a concentration evaporator (TurboVap® LV, Caliper LifeSciences, USA) with a nitrogen stream. The dried sample was reconstituted with 80 µl of 2.5% zinc sulfate (Riedel-de Häen) to precipitate the traces of remaining blood. One ml of ethyl acetate (Fluka) was added into the samples, vortexed, shaken vigorously for 10 min and centrifuged for 10 min at 17 000×g. The organic phase was separated and ethyl acetate treatment was repeated for the inorganic phase. After centrifugation, the organic phase was recovered and mixed with the previous one. The solvent was evaporated in the concentration evaporator with a nitrogen stream and the dried residue was reconstituted with 100 µl of 0.1% formic acid/ACN, 9∶1. The samples were kept frozen (−20°C) until analyzed by LC-MS.

### LC-MS

Samples were analyzed by Waters Acquity UPLC® instrument (Waters Corp., Millford, MA, USA) connected to Agilent 6410 triple-quadrupole mass spectrometer (Agilent Technologies, Santa Clara, CA, USA) using electrospray ionization (ESI). Aqueous 0.01% formic acid and ACN were used as eluents at a flow rate of 0.4 ml/min. A linear gradient elution was carried out as follows: 10–60% ACN for 0–5 min, 60–95% ACN for 5–5.1 min, 95% ACN for 5.1–7.1 min, 95–10% ACN for 7.1–7.2 min and 10% ACN for 7.2–10 min. The Waters XBridge C18 column (100×2.1 mm, 3.5 µm) and a pre-column of the similar stationary phase (10×2.1 mm, 3.5 µm) were used. The injection volume was 10 µl. Nitrogen (Parker Balston® N2-22 nitrogen generator, Parker Hannifin Corporation, Haverhill, USA) was used as a nebulizer (45 psi), curtain (10 l/min, 350°C), and collision gas. The ESI needle (4000 V) and fragmentor (90 V) voltages as well as the collision energy (CE) were optimized for DXR and ISTD. MS detection with ESI in the positive ion mode was carried out using selected reaction monitoring (SRM) with the following reactions: DXR (*m/z* 544.3→397.1 (CE 7V), 321.1 (CE 40V)) and ISTD (*m/z* 528.3→363.1 (CE 10V), 321.1 (CE 25V). Agilent Mass Hunter software version B.01.03 was used for data acquisition and processing.

### Labeling of Liposomes with ^99m^Technetium

Biotinylated liposomes for ^99m^Tc-labeling were prepared as described earlier, except 200 mM glutathione (GSH, Sigma-Aldrich) in PBS (pH 7.4) was used for hydration of the liposomes. After extrusion, the liposomes were passed through PD-10 column (Sigma-Aldrich) equilibrated with PBS. ^99m^Tc-HMPAO method for radiolabeling of GSH-liposomes was modified from the procedure described by Goins et al. [42]. Briefly, 1 ml of ^99m^Tc-pertechnetate (1.5 GBq) was added to a HMPAO-kit (Ceretec®, GE Healthcare, USA) and mixed thoroughly. After 5 min, 1 ml of ^99m^Tc-HMPAO and 1 ml of GSH-containing liposomes (15 µmol PL) were mixed and incubated for 20 min at room temperature. Radiochemical purity of ^99m^Tc-HMPAO was determined with Sep-Pak-column (Waters, USA) according to the instructions of the manufacturer. The purity was always more than 90%. ^99m^Tc-liposomes were purified by passage over a PD-10 column eluted with PBS. Liposome fractions (0.5 ml) were collected and the fractions containing radioactivity of more than 100 MBq were pooled together and used for injections. Radioactivity was measured with dose calibrator (CRC-25R, Capintec INC, USA).

### Biodistribution of ^99m^Technetium-labeled Liposomes

This animal study was approved by the Finnish National Animal Experiment Board (ESAVI-2010-05807/Ym-23) and performed in accordance with Good Laboratory Practices for Animal Research. Five-week old female C.B-17 SCID mice were purchased from Harlan (Netherlands) and quarantined for two weeks. Intraperitoneal model of ovarian cancer was established by injecting 5×10^5^ SKOV3.ip1 cells i.p. Two days before the experiment the mice were started to feed with biotin-deficient diet (Harlan) to minimize the effect of endogenous biotin on pre-targeting with biotin-avidin [43]. After 23 days from cell injections, the mice were divided into four groups, A, B, C and D (n = 4). The mice in groups A and C were injected i.p. with 200 µl of cetuximab-neutravidin (1∶1) in PBS at a dose of 20 µg cetuximab per mouse. The mice in groups B and D received i.p. injections of PBS. After 24 h, all mice received 40–80 MBq/200 µl of biot-^99m^Tc-liposomes containing 1 µmol of phospholipid. Groups A and B were injected i.v. and groups C and D i.p., respectively.

SPECT-CT imaging was performed with a four-headed small animal scanner, NanoSPECT/CT (Bioscan Inc., USA), outfitted with 1.0 mm multipinhole apertures. Mice were anesthetized with isoflurane and SPECT images were acquired 4 h and 24 h post-injection in 20 projections using time per projection of 120 s and 180 s, respectively. CT imaging was carried out with 45 kVp tube voltage in 180 projections. SPECT images were reconstructed with HiSPECT NG software (Scivis GmbH, Germany) and fused with CT datasets by using InVivoScope software (Bioscan Inc., USA).

After the 24 h time-point of liposome injections, the mice were sacrificed by cervical dislocation, blood was collected by heart puncture and tissue samples were collected in tarred tubes. Major organs and tissues were weighted and their radioactivities measured with a gamma counter (RiaCalc. WIZ, Wallac 1480 WIZARD® 3″, Finland).

### Statistical Analysis

The independent samples t-test was used for comparisons (SPSS 15.0, SPSS Inc., USA). Values of *p*<0.05 were considered as statistically significant.

## Supporting Information

Figure S1 **Flow cytometric analysis of cellular affinity shown as mean fluorescence values.** The liposomes were incubated with SKOV-3 (A) and SKOV3.ip1 (B–C) cells. In the pre-targeting group, the cells were incubated with neutravidin-cetuximab for 4 h, washed and incubated with biotin-liposomes for 2 h (A–B) or 4 h (C). In the other groups, the cells were incubated with the liposomes for 2 h (A–B) or 4 h (C).
(TIF)
