# Supplementary figures and images for: Pre-Targeting and Direct Immunotargeting of Liposomal Drug Carriers to Ovarian Carcinoma
Source: PLoS One. 2012 Jul 26;7(7):e41410. doi: 10.1371/journal.pone.0041410 (PMC3406029; doi:10.1371/journal.pone.0041410)

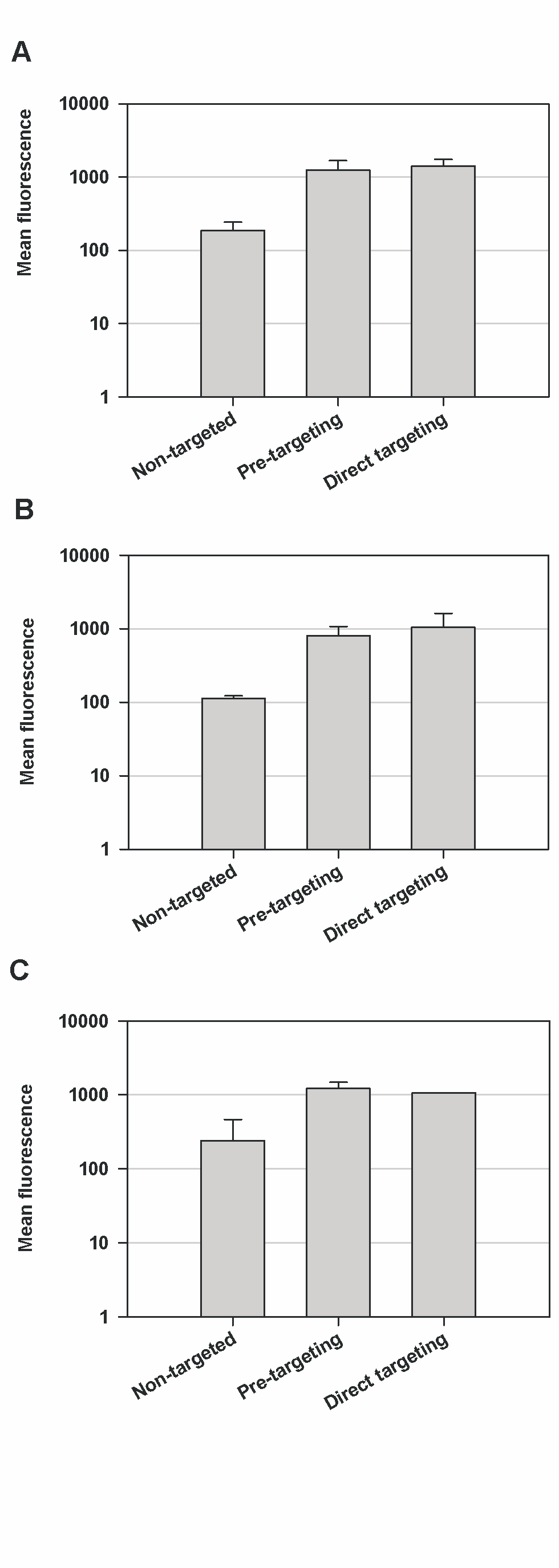

Supplement: Figure S1 — Flow cytometric analysis of cellular affinity shown as mean fluorescence values. The liposomes were incubated with SKOV-3 (A) and SKOV3.ip1 (B–C) cells. In the pre-targeting group, the cells were incubated with neutravidin-cetuximab for 4 h, washed and incubated with biotin-liposomes for 2 h (A–B) or 4 h (C). In the other groups, the cells were incubated with the liposomes for 2 h (A–B) or 4 h (C). (TIF) [file pone.0041410.s001.tif]
